# Initial treatment for FIGO 2018 stage IIIC cervical cancer based on histological type: A 14‐year multicenter study

**DOI:** 10.1002/cam4.6586

**Published:** 2023-09-28

**Authors:** Yanna Ye, Guochao Zhang, Zhiqiang Li, Biliang Chen, Hongwei Zhao, Ying Yang, Li Wang, Jilong Yao, Xiaolin Chen, Yahong Huang, Jinghe Lang, Ping Liu, Chunlin Chen

**Affiliations:** ^1^ Department of Midwifery, Faculty of Health Dongguan Polytechnic Dongguan China; ^2^ Department of Obstetrics and Gynecology, Nanfang Hospital Southern Medical University Guangzhou China; ^3^ Department of General Surgery China‐Japan Friendship Hospital Beijing China; ^4^ Department of Obstetrics and Gynecology Xijing Hospital of Airforce Medical University Xian China; ^5^ Department of Gynecologic Oncology Shanxi Provincial Cancer Hospital Taiyuan China; ^6^ Department of Obstetrics and Gynecology, Xinqiao Hospital Army Medical University Chongqing China; ^7^ Department of Gynecologic Oncology, Affiliated Cancer Hospital Zhengzhou University Zhengzhou China; ^8^ Department of Obstetrics and Gynecology Shenzhen Maternal and Child Health Hospital Shenzhen China; ^9^ Department of Obstetrics and Gynecology Peking Union Medical College Hospital Beijing China

**Keywords:** adenocarcinoma/adenosquamous cell carcinoma, cervical cancer, cervical cancer treatment, squamous cell carcinoma, stage IIIC cervical cancer

## Abstract

**Background:**

To compare the oncological outcomes of radical chemotherapy (R‐CT), abdominal radical hysterectomy (ARH), and neoadjuvant chemotherapy and radical surgery (NACT) for International Federation of Gynecology and Obstetrics (FIGO) 2018 stage IIIC cervical cancer, according to histological types: squamous cell carcinoma (SCC) and adenocarcinoma (AC)/adenosquamous cell carcinoma (ASC).

**Methods:**

A comparison of 5‐year overall survival (OS) and disease‐free survival (DFS) was performed for the SCC and AC/ASC subgroups for the three initial treatments, assessed using Kaplan–Meier and Cox proportional hazards regression analysis and validated using propensity score matching (PSM).

**Results:**

The study included 4086 patients: R‐CT, *n* = 1913; ARH, *n* = 1529; and NACT, *n* = 644. AC/ASC had a lower survival rate (63.7%) than SCC (73.6%) and a higher recurrence and mortality rate (36.3% and 26.4%, respectively). The 5‐year OS and DFS rates were different in the SCC group for R‐CT, ARH, and NACT (OS: 69.8% vs. 80.8% vs. 73.0%, *p* < 0.001; DFS: 66.7% vs. 70.7% vs. 56.4%, *p* < 0.001), also in the AC/ASC group (OS: 46.1% vs. 70.6% vs. 55.6%, *p* < 0.001; DFS: 42.7% vs. 64.6% vs. 40.8%, *p* < 0.001). As for initial treatment, survival outcomes were worse for AC/ASC treated with R‐CT and ARH than for SCC (both *p* < 0.05), with no group differences between the two treated with NACT.

**Conclusion:**

Initial treatment influences oncological prognosis for patients with FIGO 2018 stage IIIC cervical cancer. ARH is an alternative treatment for stage IIIC cervical SCC and AC/ASC, and NACT needs to be chosen with caution, moreover, R‐CT for AC/ASC requires careful selection.

## Graphical abstract

This study is focused on comparing the oncological outcomes of different treatment modalities (R‐CT, ARH, and NACT) in patients with FIGO2018 stage IIIC cervical cancer based on their histological types (SCC, AC, and ASC). This study included data from 47 hospitals in developing countries and spans a 14‐year period further highlights the potential impact of its findings on the treatment of FIGO 2018 stage IIIC cervical cancer. The findings of this study support the judicious application of treatment strategies for these patients.

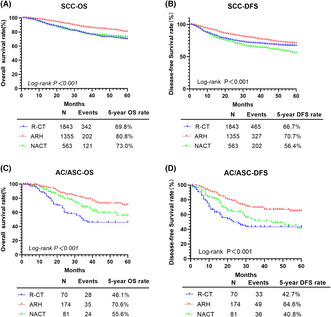

## 1 INTRODUCTION

In 2020, 604,000 new cases of cervical cancer (CC) were diagnosed, with 342,000 deaths, making CC the fourth most prevalent cancer and fourth leading cause of cancer‐related deaths among women worldwide.^1^ There are geographic differences in CC incidence and mortality rates, with the highest rates in sub‐Saharan Africa and high rates in Melanesia, South America, and Southeast Asia.^1^ Tumor staging is important to provide uniform guidance for oncological management and treatment.^2^ The International Federation of Gynecology and Obstetrics (FIGO) staging system^3^ is the most accepted tumor staging classification for CC, with major revisions in 2018 including stage IIIC. However, the clinical significance of stage IIIC CC is currently disputed as it includes all patients with lymph node metastasis (LNM), resulting in high heterogeneity and inconsistent patient prognosis within one stage.^4^, ^5^, ^6^, ^7^, ^8^, ^9^

Two initial treatments for CC are generally accepted: abdominal radical hysterectomy (ARH) and radical chemoradiation therapy (R‐CT). In recent years, however, neoadjuvant chemotherapy and radical surgery (NACT) have been used to treat CC. Nevertheless, both the FIGO 2018 guidelines^3^ and the 2023 National Comprehensive Cancer Network (NCCN) guidelines^10^ recommend only R‐CT for the initial treatment of stage IIIC CC, without proposing a strategy for graded treatment. This marks a departure from past standard medical practice in which ARH and NACT were considered as treatment options. It is important to note that the studies supporting the FIGO and NCCN recommendations for the initial treatment of stage IIIC CC were mainly published between 1999 and 2011; cases included in the analyses therefore did not fully comply with current treatment protocols. Moreover, these studies included cases with stage IIB or higher CC, both with and without LNM, with no data available for stage IIIC only. Lastly, these studies were mainly performed in developed countries in Europe and in the United States, resulting in a lack of data from less developed regions with high CC‐related morbidity and mortality.

A principal type of CC is squamous cell carcinoma (SCC), diagnosed in 75%–80% of cases, followed by adenocarcinoma (AC) diagnosed in 20%–25% of cases; adenosquamous cell carcinoma (ASC) is a rare type of CC. Previous studies have reported inconsistent prognoses for these different types of CC, with similar outcomes reported in several studies,^11^, ^12^, ^13^, ^14^, ^15^, ^16^, ^17^, ^18^, ^19^, ^20^, ^21^ while others have reported differences in prognosis.^22^, ^23^, ^24^, ^25^, ^26^, ^27^, ^28^, ^29^ In addition, various histological types of CC respond differently to initial treatment, which has an impact on prognosis.^28^, ^30^, ^31^, ^32^, ^33^ Consequently, the initial treatment for stage IIIC CC should be individualized and be specific to the histological type. Therefore, our aim in this study was to compare the oncological outcomes of R‐CT, ARH, and NACT for FIGO 2018 stage IIIC CC, according to histological type: SCC and AC/ASC. Further, our study aimed to provide a real‐world basis from less developed countries for the selection of initial treatment of stage IIIC CC.

## 2 MATERIAL AND METHODS

### 2.1 Study design and sample

For this retrospective study, the data used were obtained from the Chinese Cervical Cancer Clinical Research Database developed through a clinical trial (Project 1538; Ethics Clearance NFEC‐2017‐135; Clinical trial registration number: CHiCTR1800017778, http://apps.who.int/trialsearch/). The database includes 63,926 cases of CC, collected across 47 hospitals in China. Included are patients’ clinical information, pretreatment biopsy, laboratory and imaging information, treatment‐related information, treatment complications, and postoperative pathology. Data were collected by two gynecologists who received specific training for the clinical trial, using EpiData 3.1 (EpiData Association) for dual data entry and standard interview for follow‐up data by telephone or outpatient visits. Details of the data collection and follow‐up methods have been previously been described.^34^, ^35^

For our study, the selection criteria for eligible cases were listed below: age ≥18 years; CC detected through cervical biopsy; histology confirming SCC, AC, or ASC type of CC; FIGO 2018 stage IIIC classification. With regard to treatment, the inclusion criteria were as follows^3^, ^36^: R‐CT, external radiation therapy (RT) at a dose ≥45 Gy (1.8–2 Gy per fraction)/intra‐cavitary RT at a dose ≥40 Gy (6–7 Gy per fraction, high dose rate), and the total dose of external and intra‐cavitary RT is usually ≥85 Gy, on or off concurrent platinum‐containing chemotherapy (for at least 8 weeks); ARH, Q‐M type‐B or type‐C radical hysterectomy and pelvic lymphadenectomy, with or without para‐aortic lymphadenectomy, with postoperative adjuvant therapy; NACT, consisting of platinum‐based neoadjuvant therapy (2–3 cycles) and Q‐M type‐B or type‐C radical hysterectomy and pelvic lymphadenectomy, with or without para‐aortic lymphadenectomy, with postoperative adjuvant therapy; with follow‐up endings. Exclusion criteria consisted of violation of selection criteria, missed visits, cancer of the uterine cervix stump, and CC combined with other malignancies or pregnancy.

### 2.2 Outcomes

The outcomes for this study were overall survival (OS) and disease‐free survival (DFS), with a cutoff point of 5 years after treatment. OS is defined as the last time point from diagnosis to effective follow‐up or death from any cause. DFS is defined as the last time point from diagnosis to follow‐up, relapse, or death.

### 2.3 Statistics

Continuous variables were described by their mean ± standard deviation, with independent samples *t*‐test used for between‐group comparisons. Categorical values were described by their percentage (%), with the chi‐squared test or Fisher's exact probability test used, as appropriate, for between‐group comparisons. The Kaplan–Meier method was used for survival analysis. Independent risk factors were identified using Cox proportional hazards models, and hazard ratios (HRs) and 95% confidence intervals (95% CI) were calculated. Propensity score matching (PSM) was used to minimize the influence of baseline differences between groups. All analyses were performed using SPSS (version 29; IBM Corp.), with significance set at a *p*‐value <0.05.

## 3 RESULTS

### 3.1 Case screening results

**FIGURE 1:**
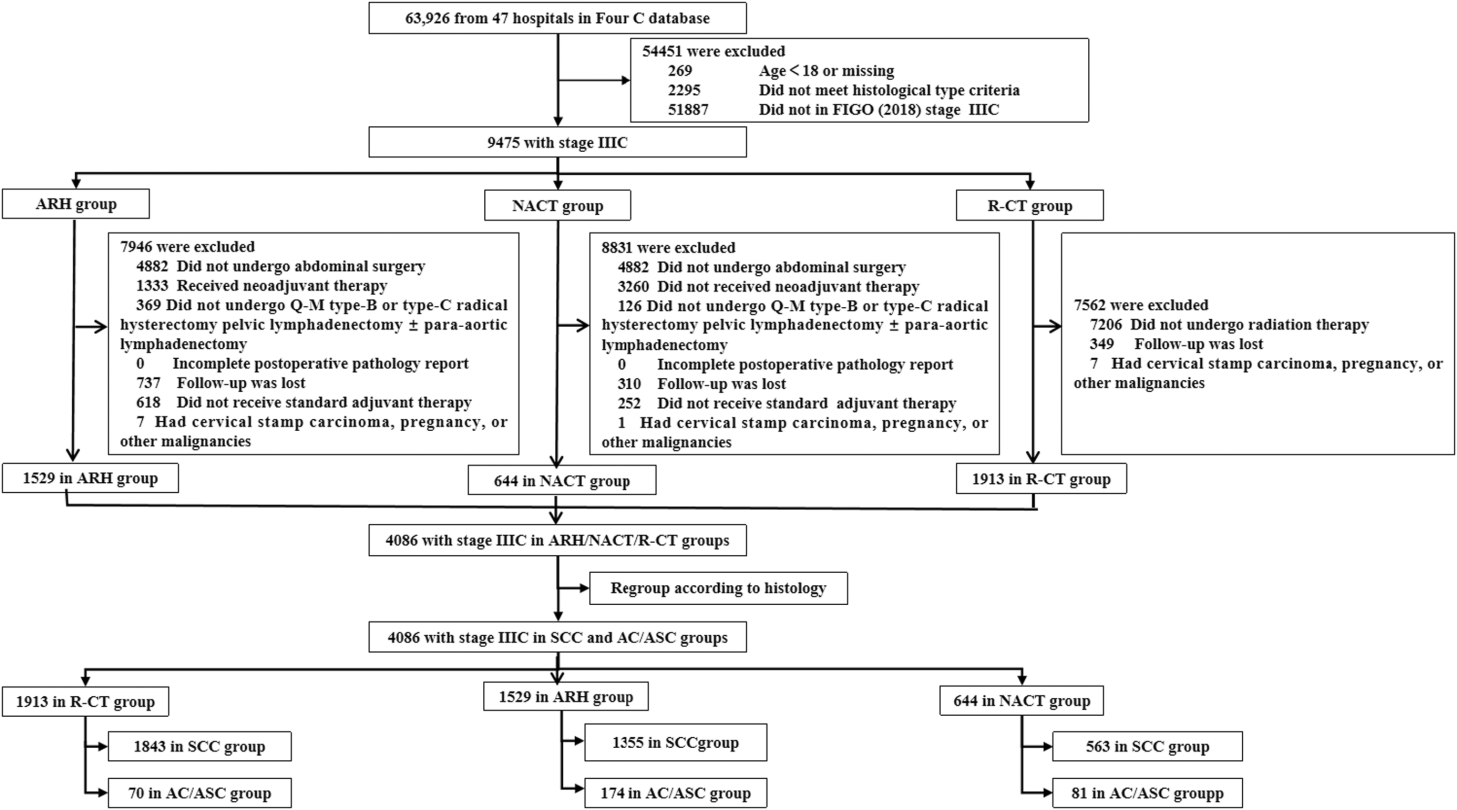
Patient selection flow diagram.

From the database, 4086 CC cases met our inclusion/exclusion criteria, with no missing values and the distribution per treatment as follows: R‐CT, *n* = 1913 (radiation therapy, *n* = 294; radiation therapy + chemotherapy, *n* = 1619); ARH, *n* = 1529; and NACT, *n* = 644. All cases in this study were combined with pelvic LNM, including 21 cases in the NACT group and 36 cases in the ARH group with para‐aortic LNM. The selection process for cases is shown in Figure 1, with baseline characteristics of patients summarized in Table S1. Baseline comparisons of patients with SCC and AC/ASC in the R‐CT, ARH, and NACT groups are shown in Tables S2–S4.

### 3.2 Survival and metastasis outcomes

Between‐group differences in survival rates are reported in Table 1. The survival rate was 73.6% for patients with SCC and 63.7% for those with AC/ASC CC, with recurrence and mortality rates of 26.4% and 36.3%, respectively (*p* < 0.001). The pelvic metastasis rate was 3.8% for patients with SCC and 6.2% for those with AC/ASC, with distant metastasis rates of 7% and 12.3%, respectively, and rates of pelvic + distant metastasis of 1.7% and 4%, respectively (all *p* < 0.001).

**TABLE 1:** Survival outcomes of patients with SCC and AC/ASC.

Variables	SCC group (*n* = 3761) %	AC/ASC group (*n* = 325) %	*p*
Survival			
Survival	2767 (73.6)	207 (63.7)	<0.001
Recurrence + mortality	994 (26.4)	118 (36.3)	
Recurrence			
Pelvic metastasis	144 (3.8)	20 (6.2)	<0.001
Distant metastasis	263 (7)	40 (12.3)	
Pelvic + distant metastasis	64 (1.7)	13 (4)	
Unknown metastasis	529 (14.1)	45 (13.8)	
No	2761 (73.4)	207 (63.7)	

Abbreviations: AC, adenocarcinoma; ASC, adenosquamous cell carcinoma; SCC, squamous cell carcinoma.

### 3.3 Comparison of oncological outcomes

**FIGURE 2:**
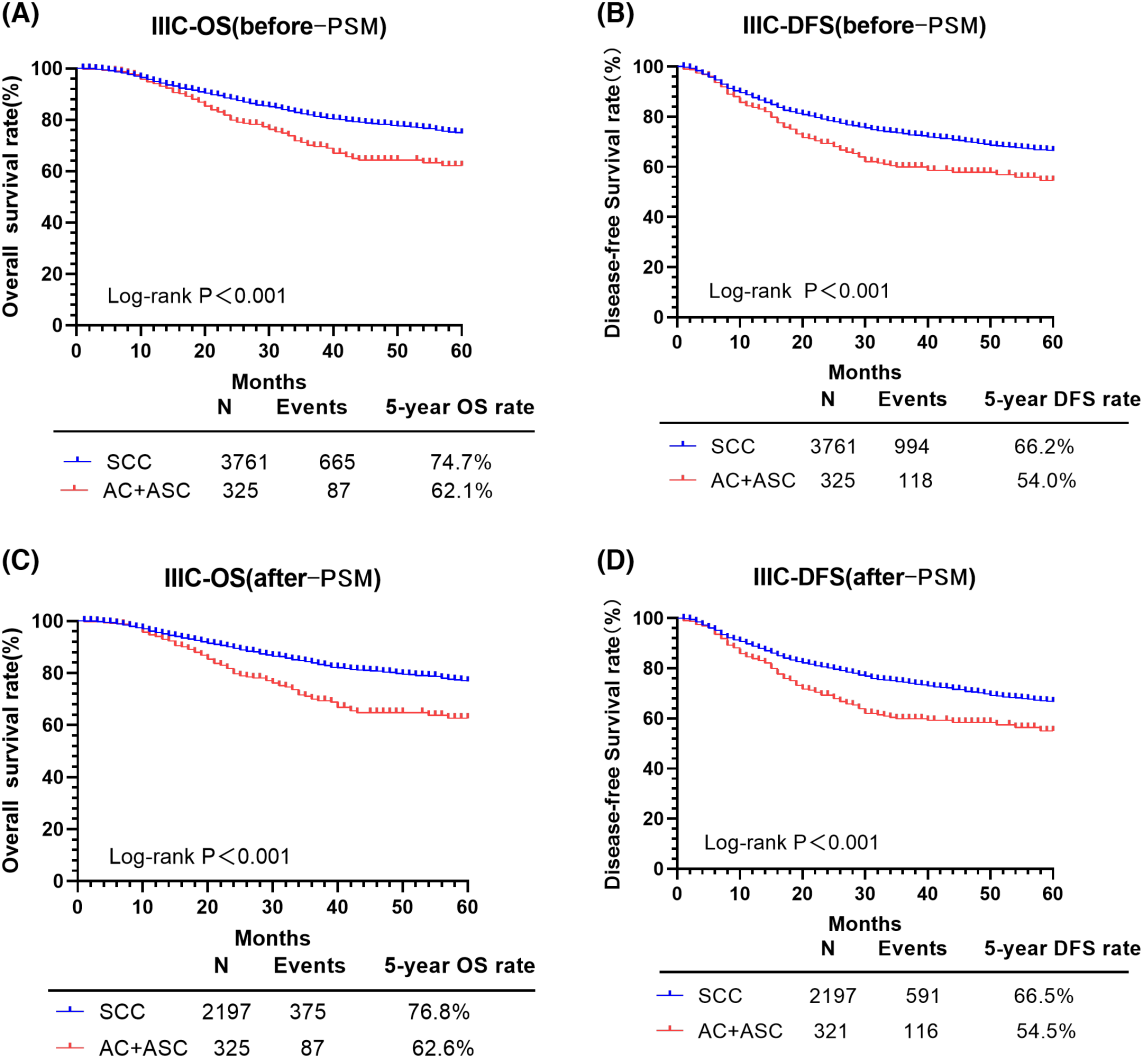
OS and DFS of patients with SCC and AC/ASC. AC, adenocarcinoma; ASC, adenosquamous cell carcinoma; DFS, disease‐free survival; OS, overall survival; PSM, propensity score matching; SCC, squamous cell carcinoma.

The 5‐year OS and DFS as between the SCC group and the AC/ASC group are shown in Figure 2. After PSM, the 5‐year OS was 76.8% for the SCC group and 62.6% for the AC/ASC group (*p* < 0.001), with a DFS of 66.5% and 54.5%, respectively (*p* < 0.001). Overall, compared to the SCC group, survival was significantly lower for the AC/ASC group, after PSM: OS: HR = 1.851, 95% CI: 1.464–2.341, *p* < 0.001; and DFS: HR = 1.582, 95% CI: 1.296–1.931, *p* < 0.001.

**FIGURE 3:**
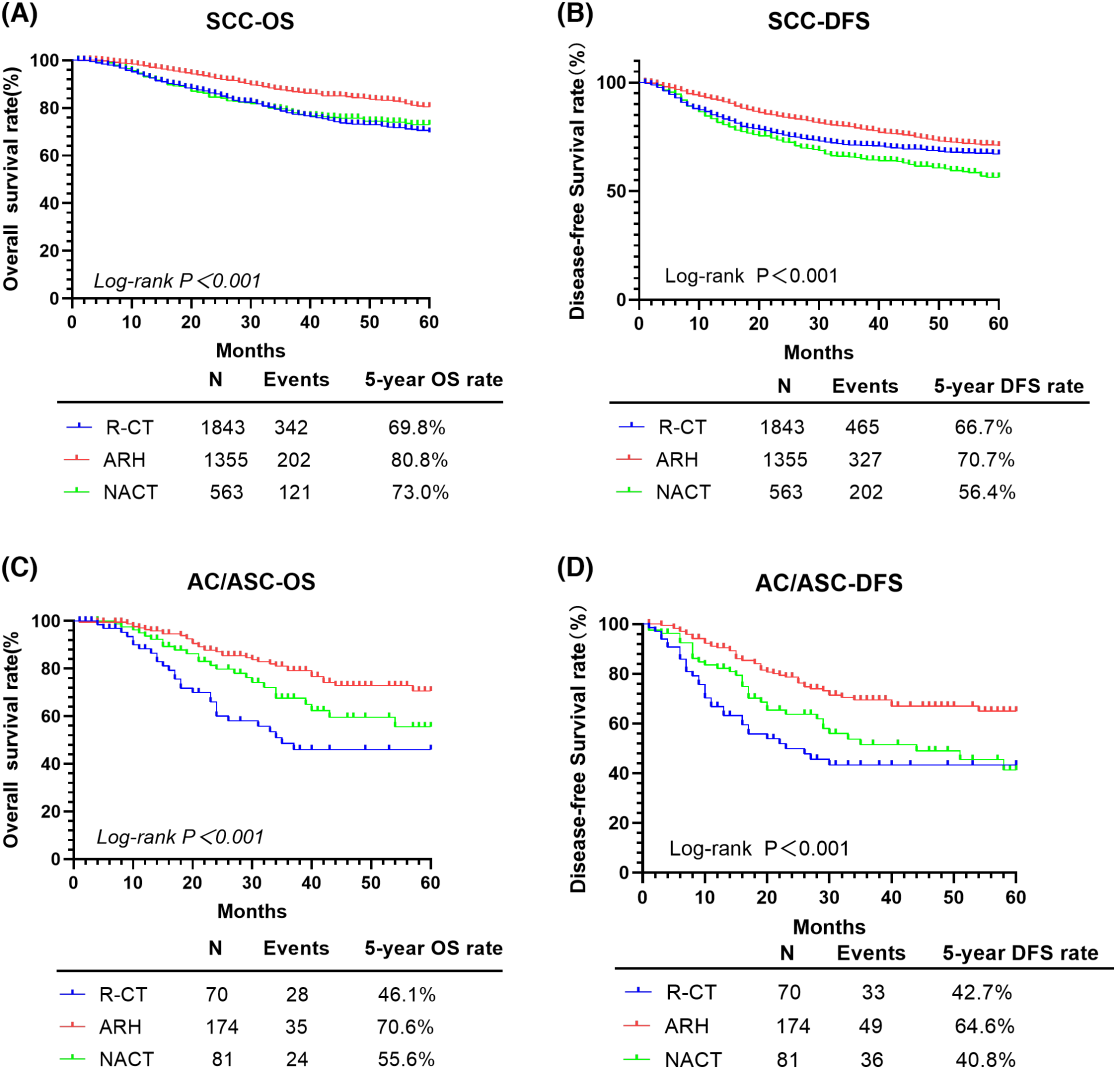
OS and DFS of patients with SCC and AC/ASC in the R‐CT, ARH and NACT groups. AC, adenocarcinoma; ARH, abdominal radical hysterectomy; ASC, adenosquamous cell carcinoma; DFS, disease‐free survival; NACT, neoadjuvant chemotherapy and radical surgery; OS, overall survival; R‐CT, radical chemoradiotherapy; SCC, squamous cell carcinoma.

Regarding initial treatment (Figure 3), the 5‐year OS and DFS rates were different for R‐CT, ARH, and NACT for the SCC group, respectively, as follows: OS: 69.8% versus 80.8% versus 73.0%, *p* < 0.001; and DFS: 66.7% versus 70.7% versus 56.4%, *p* < 0.001. Differences were also identified between these three initial treatments for the AC/ASC group, as follows: OS: 46.1% versus 70.6% versus 55.6%, *p* < 0.001; and DFS: 42.7% versus 64.6% versus 40.8%, *p* < 0.001. Compared to R‐CT, survival was significantly better for ARH, after PSM: OS: HR = 0.569, 95% CI: 0.452–0.717, *p* < 0.001; and DFS: HR = 0.677, 95% CI: 0.558–0.822, *p* < 0.001. Survival rates did not differ between the R‐CT and NACT groups, after PSM: OR: HR = 0.909, 95% CI: 0.697–1.184, *p* = 0.478; and DFS: HR = 1.165, 95% CI: 0.938–1.447, *p* = 0.167). After PSM, age did not influence either the 5‐year OS (*p* = 0.162) or DFS (*p* = 0.703; Table 2).

**TABLE 2:** Cox multivariate survival analysis of patients with FIGO 2018 stage IIIC CC.

Variables	Before PSM						After PSM					
	5‐year OS			5‐year DFS			5‐year OS			5‐year DFS		
	HR	95% CI	*p*	HR	95% CI	*p*	HR	95% CI	*p*	HR	95% CI	*p*
Age (years)	1.007	1.000–1.015	0.043	1.001	0.995–1.007	0.776	1.007	0.997–1.017	0.162	1.002	0.994–1.010	0.703
Histological type												
SCC	1 (Ref)	—	—	1	—	—	1 (Ref)	—	—	1	—	—
AC/ASC	1.843	1.468–2.313	<0.001	1.594	1.313–1.935	<0.001	1.851	1.464–2.341	<0.001	1.582	1.296–1.931	<0.001
Initial treatment modality			<0.001			<0.001			<0.001			<0.001
R‐CT	1 (Ref)	—	—	1	—	—	1 (Ref)	—	—	1	—	—
ARH	0.569	0.479–0.675	<0.001	0.696	0.605–0.802	0.026	0.569	0.452–0.717	<0.001	0.677	0.558–0.822	<0.001
NACT	0.947	0.772–1.160	0.598	1.204	1.022–1.418	<0.001	0.909	0.697–1.184	0.478	1.165	0.938–1.447	0.167

Abbreviations: AC, adenocarcinoma; ARH, abdominal radical hysterectomy; ASC, adenosquamous cell carcinoma; CC, cervical cancer; CI, confidence interval; DFS, disease‐free survival; FIGO, International Federation of Gynecology and Obstetrics; HR, hazard ratio; NACT, neoadjuvant chemotherapy and radical surgery; OS, overall survival; PSM, propensity score matching; R‐CT, radical chemoradiotherapy; SCC, squamous cell carcinoma.

Differences in survival by treatment and histological type are reported in Table 3. For the SCC type, survival was better with ARH than R‐CT as an initial treatment, OS: HR = 0.589, 95% CI: 0.491–0.706, *p* < 0.001; and DFS: HR = 0.725, 95% CI: 0.626–0.840, *p* < 0.001. By contrast, for NACT as compared to R‐CT, the 5‐year DFS was worse (HR = 1.245, 95% CI: 1.048–1.480, *p* < 0.001) but not the 5‐year OS (HR = 0.982, 95% CI: 0.790–1.220, *p* = 0.870). Age did not influence the 5‐year OS (*p* = 0.099) or DFS (*p* = 0.963). For the AC/ASC type, survival was again better for ARH than R‐CT, OS: HR = 0.399, 95% CI: 0.236–0.676, *p* = 0.001; and DFS: HR = 0.425, 95% CI: 0.267–0.678, *p* < 0.001. For this type, there was no influence of NACT on either the 5‐year OS (*p* = 0.180) or DFS (*p* = 0.340). Age did not influence the 5‐year OS (*p* = 0.174) or DFS (*p* = 0.500).

**TABLE 3:** Cox multivariate survival analysis of patients with SCC and AC/ASC.

Variables	SCC group						AC/ASC group					
	5‐year OS			5‐year DFS			5‐year OS			5‐year DFS		
	HR	95% CI	*p*	HR	95% CI	*p*	HR	95% CI	*p*	HR	95% CI	*p*
Age (years)	1.006	0.999–1.014	0.099	1.000	0.994–1.007	0.963	1.015	0.993–1.037	0.174	1.006	0.988–1.025	0.500
Initial treatment modality			<0.001			<0.001			0.002			<0.001
R‐CT	1 (Ref)	—	—	1	—	—	1 (Ref)	—	—	1	—	—
ARH	0.589	0.491–0.706	<0.001	0.725	0.626–0.840	<0.001	0.399	0.236–0.676	0.001	0.425	0.267–0.678	<0.001
NACT	0.982	0.790–1.220	0.870	1.245	1.048–1.480	<0.001	0.674	0.379–1.200	0.180	0.783	0.473–1.295	0.340

Abbreviations: AC, adenocarcinoma; ARH, abdominal radical hysterectomy; ASC, adenosquamous cell carcinoma; CI, confidence interval; DFS, disease‐free survival; HR, hazard ratio; NACT, neoadjuvant chemotherapy and radical surgery; OS, overall survival; PSM, propensity score matching; R‐CT, radical chemoradiotherapy; SCC, squamous cell carcinoma.

### 3.4 Comparison of oncological outcomes between the SCC and AC/ASC type for the R‐CT treatment

**FIGURE 4:**
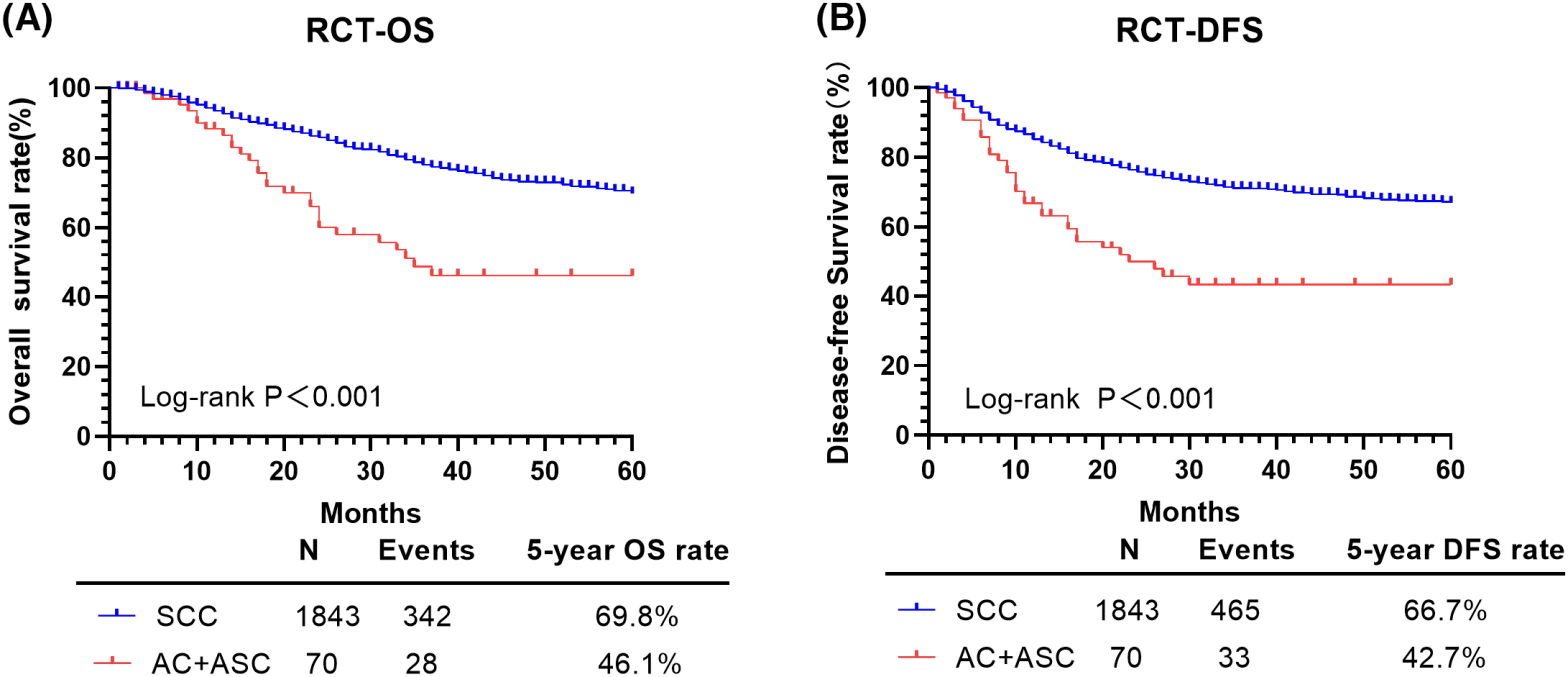
OS and DFS of patients with SCC and AC/ASC in the R‐CT group. AC, adenocarcinoma; ASC, adenosquamous cell carcinoma; DFS, disease‐free survival; OS, overall survival; R‐CT, radical chemoradiotherapy; SCC, squamous cell carcinoma.

When considering only R‐CT treatment, survival was better for the SCC than AC/ASC groups, respectively, as follows (Figure 4; Table 4): OS: 69.8% versus 46.1%, *p* < 0.001; and DFS: 66.7% versus 42.7%, *p* < 0.001. For this group, PSM was not required as between‐group differences baseline characteristics were flat. The HRs for lower survival for the AC/ASC than SCC type were as follows: OS: 1.667, 95% CI: 1.164–2.388, *p* = 0.005; and DFS: 1.402, 95% CI: 1.038–1.894, *p* = 0.028. There was no effect of age on either the 5‐year OS (*p* = 0.173) or DFS (*p* = 0.626).

**TABLE 4:** Cox multivariate survival analysis of patients with SCC and AC/ASC in the R‐CT group.

Variables	5‐year OS			5‐year DFS		
	HR	95% CI	*p*	HR	95% CI	*p*
Age (years)	1.009	0.996–1.023	0.173	1.003	0.992–1.014	0.626
Histological type						
SCC	1 (Ref)	—	—	1 (Ref)	—	—
AC/ASC	1.667	1.164–2.388	0.005	1.402	1.038–1.894	0.028

Abbreviations: AC, adenocarcinoma; ASC, adenosquamous cell carcinoma; CI, confidence interval; DFS, disease‐free survival; HR, hazard ratio; OS, overall survival; SCC, squamous cell carcinoma.

### 3.5 Comparison of the oncological outcomes between the SCC and AC/ASC types for the ARH treatment

**FIGURE 5:**
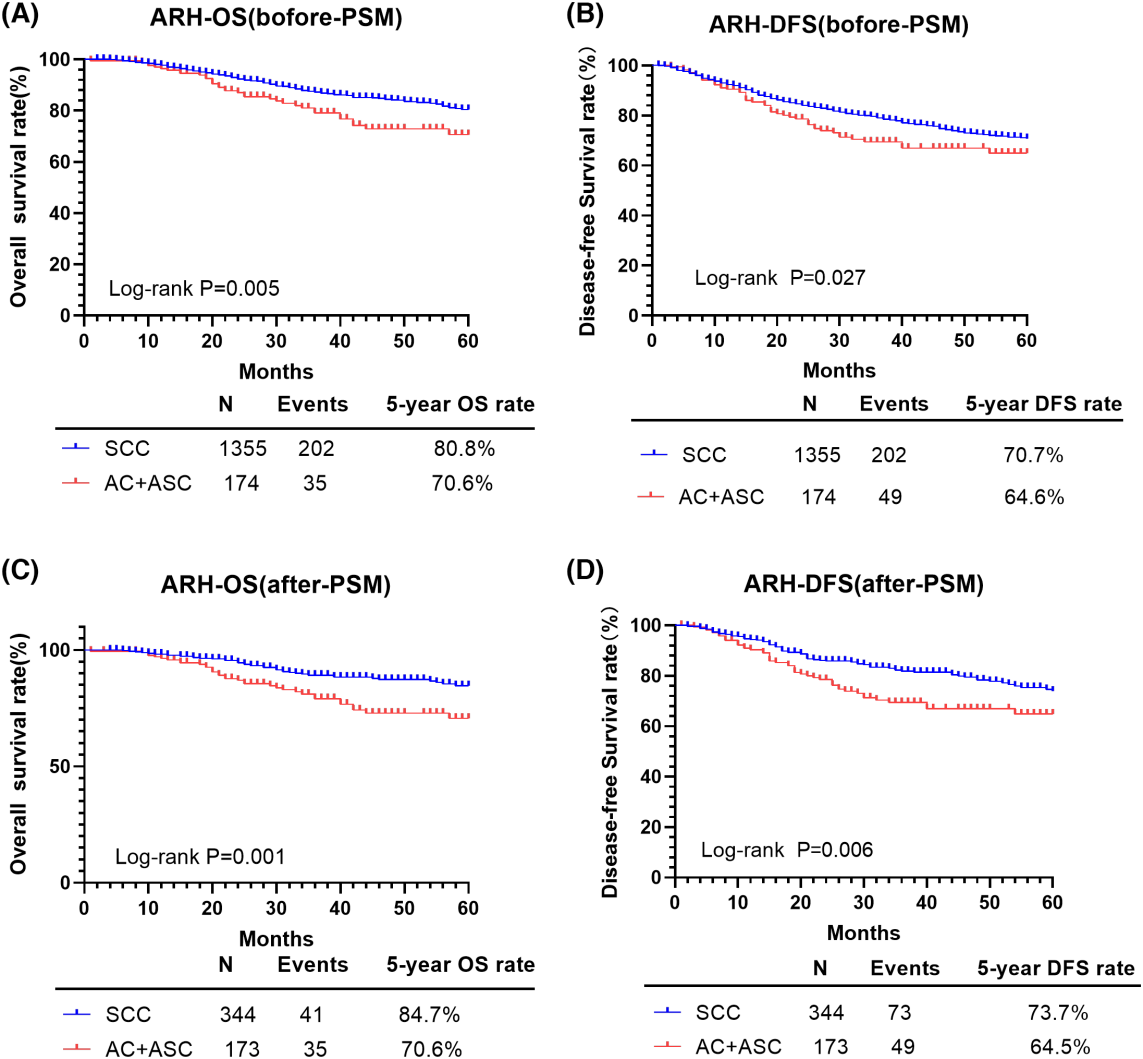
OS and DFS of patients with SCC and AC/ASC in the ARH group. AC, adenocarcinoma; ARH, abdominal radical hysterectomy; ASC, adenosquamous cell carcinoma; DFS, disease‐free survival; OS, overall survival; PSM, propensity score matching; R‐CT, radical chemoradiotherapy; SCC, squamous cell carcinoma.

When considering only ARH treatment, the Kaplan–Meier analysis revealed statistically sharp differences in survival between the SCC than AC/ASC groups, respectively, as follows (Figure 5; Table 5), after PSM: OS: 84.7% versus 70.6%, *p* = 0.001; and DFS: 73.7% versus 64.5%, *p* = 0.006. The HRs for lower survival for the AC/ASC than SCC type were as follows, after PSM: OS: 2.210, 95% CI: 1.399–3.493, *p* = 0.001; and DFS: 1.717, 95% CI: 1.191–2.475, *p* = 0.004. Additionally, compared to cervical stromal invasion ≤1/2, the cervical stromal invasion >1/2 was associated with a worse 5‐year OS (*p* = 0.010) and DFS (*p* = 0.003) after PSM, with an unknown cervical stromal invasion being associated with a worse 5‐year OS (*p* = 0.019) but not DFS (*p* = 0.090). Parametrial involvement was associated with a worse 5‐year OS (*p* = 0.016) and DFS (*p* = 0.012). There were no effects of age, hysterectomy type, tumor diameter, lymphovascular space invasion (LVSI), vaginal margin, or para‐aortic lymph node (LN) with either 5‐year OS or DFS (*p* > 0.05).

**TABLE 5:** Cox multivariate survival analysis of patients with SCC and AC/ASC in the ARH group.

Variables	Before PSM						After PSM					
	5‐year OS			5‐year DFS			5‐year OS			5‐year DFS		
	HR	95% CI	*p*	HR	95% CI	*p*	HR	95% CI	*p*	HR	95% CI	*p*
Age (years)	1.001	0.992–1.019	0.431	1.000	0.989–1.011	0.974	0.989	0.966–1.013	0.364	0.985	0.967–1.004	0.117
Histological type												
SCC	1 (Ref)	—	—	1 (Ref)	—	—	1 (Ref)	—	—	1 (Ref)	—	—
AC/ASC	1.825	1.268–2.627	<0.001	1.485	1.095–2.013	0.011	2.210	1.399–3.493	0.001	1.717	1.191–2.475	0.004
Hysterectomy type			0.317			0.510			0.356			0.492
Type QM‐B	1 (Ref)	—	—	1 (Ref)	—	—	1 (Ref)	—	—	1 (Ref)	—	—
Type QM‐C1	1.244	0.938–1.650	0.130	1.143	0.909–1.436	0.252	1.420	0.880–2.292	0.151	1.266	0.858–1.869	0.234
Type QM‐C2	0.00	0–1.440E+101	0.937	1.199	0.294–4.889	0.800	0.00	0–5.996E+169	0.963	0.00	0–2.294E+138	0.954
Tumor diameter (cm)			0.596			0.227			0.725			0.397
≤4	1 (Ref)	—	—	1 (Ref)	—	—	1 (Ref)	—	—	1 (Ref)	—	—
>4	1.102	0.835–1.453	0.493	1.169	0.939–1.456	0.162	1.215	0.749–1.972	0.430	1.297	0.887–1.895	0.179
Unknown	1.310	0.699–2.453	0.399	1.359	0.820–2.250	0.234	1.006	0.352–2.875	0.991	1.021	0.433–2.404	0.963
LVSI												
Negative	1 (Ref)	—	—	1 (Ref)	—	—	1 (Ref)	—	—	1 (Ref)	—	—
Positive	1.521	1.171–1.975	0.002	1.235	1.004–1.519	0.046	1.251	0.753–2.077	0.387	1.015	0.672–1.534	0.944
Cervical stromal invasion			0.002			<0.001			0.033			0.011
≤1/2	1 (Ref)	—	—	1 (Ref)	—	—	1 (Ref)	—	—	1 (Ref)	—	—
>1/2	2.641	1.528–4.565	0.001	2.202	1.480–3.277	0.151	6.463	1.576–26.503	0.010	3.490	1.527–7.978	0.003
Unknown	2.394	0.998–5.744	0.051	1.679	0.828–3.406	0.001	7.226	1.381–37.802	0.019	1.679	0.858–8.416	0.090
Parametrial involvement												
Negative	1 (Ref)	—	—	1 (Ref)	—	—	1 (Ref)	—	—	1 (Ref)	—	—
Positive	0.644	0.388–1.070	0.089	0.541	0.373–0.786	<0.001	2.547	1.193–5.435	0.016	2.251	1.197–4.233	0.012
Vaginal margin												
Negative	1 (Ref)	—	—	1 (Ref)	—	—	1 (Ref)	—	—	1 (Ref)	—	—
Positive	1.061	0.554–2.034	0.858	1.231	0.763–1.985	0.395	1.944	0.741–5.102	0.177	1.227	0.486–3.102	0.665
Para‐aortic lymph node			0.327			0.182			0.357			0.565
Negative	1 (Ref)	—	—	1 (Ref)	—	—	1 (Ref)	—	—	1 (Ref)	—	—
Positive	1.832	0.771–4.355	0.171	1.794	0.965–3.337	0.065	2.124	0.508–8.874	1.295	1.384	0.411–4.078	0.659
Unknown	1.317	0.832–2.082	0.240	1.155	0.825–1.617	0.400	1.837	0.776–4.345	1.384	1.717	0.763–2.512	0.285

Abbreviations: AC, adenocarcinoma; ARH, abdominal radical hysterectomy; ASC, adenosquamous cell carcinoma; CI, confidence interval; DFS, disease‐free survival; HR, hazard ratio; LVSI, lymphovascular space invasion; OS, overall survival; PSM, propensity score matching; SCC, squamous cell carcinoma.

### 3.6 Comparison of oncological outcomes between the SCC and AC/ASC types for the NACT treatment

**FIGURE 6:**
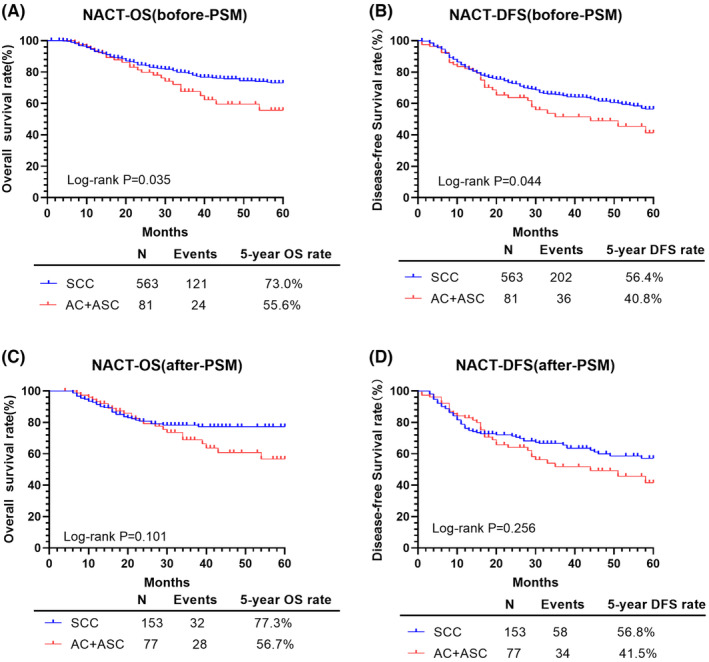
OS and DFS of patients with SCC and AC/ASC in the NACT group. AC, adenocarcinoma; ASC, adenosquamous cell carcinoma; DFS, disease‐free survival; NACT, neoadjuvant chemotherapy and radical surgery; OS, overall survival; PSM, propensity score matching; SCC, squamous cell carcinoma.

For NACT treatment, there was no difference in survival between the SCC and AC/ASC types, respectively, after PSM (Figure 6; Table 6): OS: 77.3% versus 56.7%, *p* = 0.101; and DFS: 56.8% versus 41.5%, *p* = 0.256. This absence of a difference in survival between the two types was confirmed on Cox multivariate analysis, with HRs of survival for the AC/ASC compared to SCC type, after PSM, as follows: OS: 10,154.21, 95% CI: 0.00–6.065e+68, *p* = 0.904; and DFS: 7302.950, 95% CI 0.00–2.364e+51, *p* = 0.873. However, compared with a tumor diameter of ≤4 cm, a tumor diameter >4 cm was associated with a worse 5‐year OS, after PSM (*p* = 0.040) but not DFS (*p* = 0.299). Furthermore, compared with a cervical stromal invasion of ≤1/2, a cervical stromal invasion >1/2 was associated with a worse 5‐year DFS (*p* = 0.022) but not OS (*p* = 0.199), with no effect of an unknown cervical stromal invasion on either the 5‐year OS (*p* = 0.704) or DFS (*p* = 0.873). Parametrial involvement was associated with a worse 5‐year DFS (*p* = 0.004) but not OS (*p* = 0.302). Lastly, compared to negative para‐aortic LN, positive para‐aortic LN was associated with a worse 5‐year DFS (*p* = 0.022) but not OS (*p* = 0.204), with no effect of unknown para‐aortic LN on either the 5‐year OS or DFS (both *p* > 005). There were no effects of age, hysterectomy type, LVSI, or vaginal margin on either the 5‐year OS or DFS (*p* > 0.05).

**TABLE 6:** Cox multivariate survival analysis of patients with SCC and AC/ASC in the NACT group.

Variables	Before PSM						After PSM					
	5‐year OS			5‐year DFS			5‐year OS			5‐year DFS		
	HR	95% CI	*p*	HR	95% CI	*p*	HR	95% CI	*p*	HR	95% CI	*p*
Age (years)	1.018	0.998–1.039	0.071	1.003	0.987–1.018	0.746	1.011	0.978–1.046	0.508	0.999	0.974–1.025	0.960
Histological type												
SCC	1 (Ref)	—	—	1 (Ref)	—	—	1 (Ref)	—	—	1 (Ref)	—	—
AC/ASC	1.501	0.959–2.348	0.075	1.273	0.884–1.834	0.195	10,154.21	0–6.065E+68	0.904	7302.950	0–2.364E+51	0.873
Hysterectomy type			0.710			0.510						
Type QM‐B	1 (Ref)	—	—	1 (Ref)	—	—	1 (Ref)	—	—	1 (Ref)	—	—
Type QM‐C1	0.00	0–4.420E+153	0.956	0.00	0–1.640E+117	0.943	1.200	0.643–2.239	0.566	1.071	0.655–1.752	0.784
Type QM‐C2	0.855	0.589–1.240	0.409	0.931	0.700–1.239	0.624	—	—	—	—	—	—
Tumor diameter (cm)			0.502			0.355			0.100			0.309
≤4	1 (Ref)	—	—	1 (Ref)	—	—	1 (Ref)	—	—	1 (Ref)	—	—
>4	1.201	0.832–1.732	0.328	1.042	0.784–1.386	0.776	1.950	1.033–3.684	0.040	1.276	0.806–2.021	0.299
Unknown	1.341	0.753–2.390	0.319	1.363	0.884–2.101	0.161	1.107	0.312–3.926	0.875	0.651	0.227–1.868	0.425
LVSI												
Negative	1 (Ref)	—	—	1 (Ref)	—	—	1 (Ref)	—	—	1 (Ref)	—	—
Positive	1.091	0.761–1.565	0.634	0.847	0.630–1.139	0.271	0.906	0.483–1.700	0.760	0.717	0.429–1.198	0.204
Cervical stromal invasion			0.217			0.093			0.277			0.029
≤1/2	1 (Ref)	—	—	1 (Ref)	—	—	1 (Ref)	—	—	1 (Ref)	—	—
>1/2	1.479	0.904–2.421	0.120	1.533	1.041–2.256	0.030	1.841	0.726–4.674	0.199	2.497	1.143–5.454	0.022
Unknown	1.022	0.425–2.459	0.961	1.331	0.693–2.558	0.391	0.656	0.074–5.793	0.704	0.878	0.178–4.333	0.873
Parametrial involvement												
Negative	1 (Ref)	—	—	1 (Ref)	—	—	1 (Ref)	—	—	1 (Ref)	—	—
Positive	1.603	0.930–2.764	0.089	1.837	1.233–2.759	0.003	1.536	0.680–3.470	0.302	2.349	1.304–4.231	0.004
Vaginal margin												
Negative	1 (Ref)	—	—	1 (Ref)	—	—	1 (Ref)	—	—	1 (Ref)	—	—
Positive	0.342	0.084–1.392	0.134	1.306	0.702–2.431	0.399	9653.501	0–6.581E+102	0.894	0.630	0.243–1.629	0.340
Para‐aortic lymph node			0.918			0.34			0.443			0.071
Negative	1 (Ref)	—	—	1 (Ref)	—	—	1 (Ref)	—	—	1 (Ref)	—	—
Positive	0.792	0.263–2.391	0.680	1.775	0.925–3.404	0.084	1.791	0.729–4.403	0.204	2.383	1.135–5.007	0.022
Unknown	0.964	0.584–1.592	0.887	0.845	0.584–1.224	0.373	8226.150	0–4.910E+68	0.906	8200.207	0‐2652E+51	0.872

Abbreviations: AC, adenocarcinoma; ASC, adenosquamous cell carcinoma; CI, confidence interval; DFS, disease‐free survival; HR, hazard ratio; LVSI, lymphovas; NACT, neoadjuvant chemotherapy and radical surgery; OS, overall survival; PSM, propensity score matching; SCC, squamous cell carcinoma.

## 4 DISCUSSION

The main findings of our comparison of oncological outcomes for the SCC and AC/ASC types of FIGO 2018 stage IIIC CC for R‐CT, ARH, and NACT as an initial treatment were as follows. First, 5‐year survival was worse for AC/ASC than SCC CC, which may result from a greater risk of recurrence and metastasis for the AC/ASC. With regard to initial treatment, in the overall comparison, after PSM, the oncological outcome of R‐CT was not better than that of ARH and was comparable with that of NACT; for AC/ASC, the same results were obtained, but for SCC, the oncological outcome of R‐CT was better than that of NACT and not better than that of ARH. When stratified by treatment type, R‐CT and ARH had better therapeutic efficacy for SCC than AC/ASC CC. Of note, the therapeutic efficacy of NACT was consistent for SCC and AC/ASC CC. Overall, the findings suggest that the survival outcome of AC/ASC was inferior to that of SCC; that ARH was an alternative treatment for SCC and AC/ASC, while NACT should be used with caution, and that R‐CT was not beneficial in treating AC/ASC.

Previous studies have reported comparable prognoses of SCC and AC/ASC types of CC,^11^, ^12^, ^13^, ^14^, ^15^, ^16^, ^17^, ^18^, ^19^, ^20^, ^21^ as well as for AC and ASC.^21^ Recently, differences in oncological characteristics, including pathophysiology, epidemiology, patterns of tumor metastasis, and prognosis, have been reported between the different histological types of CC.^22^, ^23^, ^24^, ^25^, ^26^, ^27^, ^28^, ^29^ The presence or absence of LNM might influence the outcomes, with comparable prognoses for SCC and AC types of CC without LNM but with a worse prognosis for AC than SCC type with LNM.^37^, ^38^ In our study sample of 3762 patients with SCC and 325 with AC/ASC type of FIGO 2018 stage IIIC CC, both the 5‐year OS and DFS were better for those with SCC, in alignment with the results of Nakanishi et al.^37^ and of Kodama et al.^38^

With regard to R‐CT, chemotherapy included simultaneously with radiotherapy provides a sensitizing effect on radiotherapy which may further reduce the repair capacity of tumor cells. The effects of R‐CT might be influenced by the histological types of CC. In their study of FIGO stageIB2‐IVA CC treated with concurrent chemoradiotherapy (CCRT), Zhang et al.^28^ reported lower OS for AC than SCC CC (HR: 2.10, 95% CI: 1.79–2.46, *p* < 0.0001), with higher local (HR: 1.79, 95% CI: 1.35–2.37, *p* < 0.0001) and distant (HR: 1.97, 95% CI: 1.54–2.53, *p* < 0.0001) metastasis rates. In their study, Hsieh et al.^30^ evaluated 126 patients with cervical tumors ≥4 cm under intensity‐modulated radiotherapy together with endovenous‐brachytherapy. They reported treatment efficacy for SCC CC, although negative side effects were noted, including acute hematological toxicity and advanced toxicity, such as rectal inflammation, urethral stricture, and radiation dermatitis. However, for AC/ASC CC, the R‐CT treatment was ineffective, with worse 5‐year OS, survival without local recurrence and survival without distant metastases than for SCC CC (*p* < 0.05). This was consistent with the findings of Meng et al.,^31^ who reported significantly lower survival for patients with AC than SCC, receiving CCRT (HR = 1.14, 95% CI: 1.03–1.27, *p* < 0.01). In our study, for SCC CC, the 5‐year OS was lower for R‐CT (69.8%) than ARH (80.8%) and NACT (73.0%), and the 5‐year DFS for R‐CT (66.7%) was higher than NACT (56.4%) but lower than ARH (70.7%); ARH was associated with better 5‐year OS and DFS than R‐CT, and NACT was associated with poorer DFS. Interestingly, for AC/ASC CC, R‐CT had the lowest 5‐year OS of 46.1% and a low 5‐year DFS of 42.7%, which was very close to the lowest DFS of 40.8% for NACT; ARH was associated with better 5‐year OS and DFS than R‐CT as well. However, both the 5‐year OS and DFS were significantly worse for the AC/ASC than SCC CC treated by R‐CT. Therefore, the R‐CT did not provide a therapeutic advantage overall compared to ARH or NACT; its effectiveness was better for SCC than AC/ASC CC. Considering that patients whose initial treatment was R‐CT did not undergo surgery and lacked information on risk factors such as LVSI and parametrial involvement, the therapeutic efficacy of R‐CT needs to be further confirmed in prospective studies.

Abdominal radical hysterectomy allows resection of the tumor as well as pelvic±para‐aortic LNs to clarify the status of LN involvement, which has an important role in guiding subsequent treatment and avoiding overtreatment. Yasuda et al.^39^ reported comparable 5‐year OS rates for patients treated by ARH for stage 1B1 cervical SCC (94%), AC (92.4%), and ASC (82.4%), without difference between types (*p* > 0.05). Of note, this study included patients with both positive and negative LN. By comparison, in their assessment of 92 patients with invasive CC treated by ARH, Hopkins et al.^40^ identified a significantly lower 5‐year OS for the AC than SCC CC (80% vs. 41%, *p* = 0.0001). Similar conclusions were reached in this study, both the 5‐year OS and DFS of AC/ASC CC were significantly inferior to those of SCC treated by ARH. Nevertheless, the 5‐year OS and DFS of ARH for SCC and AC/ASC CC were higher than those of R‐CT and NACT. ARH was effective in treating both SCC and AC/ASC CC, and the survival was better for SCC.

For NACT, the chemotherapeutic drugs used can directly penetrate the cell membrane to produce toxic effects on tumor cells, such as inhibition of their self‐repair and proliferation. This chemotherapeutic effect can reduce tumor size. Moreover, direct entry of these drugs into lymphatic vessels and capillaries in the pelvis can eliminate tumor cells, reducing the risk for tumor metastasis. However, the effectiveness of NACT in improving the prognosis of patients with CC has been debated.^11^, ^41^, ^42^, ^43^ He et al.^44^ performed a meta‐analysis encompassing 1559 patients with CC and found no short‐term treatment effect of NACT for SCC and AC/ASC types CC; however, a higher 5‐year OS and DFS for SCC than AC/ASC for patients with stage IIB or higher CC (OS:HR = 1.47, 95% CI: 1.06–2.06, and DFS:HR = 1.96, 95% CI: 1.61–2.38). Of note, they used the earlier FIGO tumor staging system, including patients with and without LNM. In our study, the 5‐year OS of NACT was higher than that of R‐CT, but remained lower than that of ARH (all *p* < 0.001), the 5‐year DFS was lower than ARH and R‐CT, in both SCC and AC/ASC groups. Of note, compared to R‐CT, NACT was associated with a worse DFS in the SCC group (*p* < 0.001), whereas there was no statistical difference with that in the AC/ASC group. In the subgroup analysis with PSM, there were no differences in oncologic outcomes between the SCC and AC/ASC groups treated with NACT. The inclusion in our study of patients with just LNM likely explains differences between our results and those of He et al.^44^ Overall, our findings indicated that NACT did not work well for SCC and AC/ASC, and it was consistent in treating stage IIIC cervical SCC and AC/ASC.

We found that ARH had better therapeutic efficiency in curing stage IIIC cervical SCC and AC/ASC, and NACT was less effective, while R‐CT offered no benefit in treating AC/ASC. This can be explained as follows. First, compared to SCC, early AC/ASC is prone to LNM, ovarian, and distant metastases, leading to a poor prognosis.^19^, ^20^, ^38^, ^39^, ^45^ This was consistent with our findings of higher metastasis rates for AC/ASC than for SCC type CC (36.3% vs. 26.4%, respectively), including a higher rate of pelvic metastasis (6.2% vs. 3.8%, respectively), distant metastasis (12.3% vs. 7%, respectively), and pelvic ± distant metastasis (4% vs. 1.7%, respectively). In addition, all patients treated with ARH initially in our study received standardized adjuvant therapy after surgery. As ARH allows for resection of tumor tissue, underlying pelvic and abdominal tissues, and metastatic LN, it reduces the tumor load while clarifying the indications for postoperative adjuvant therapy. Timely and standardized adjuvant therapy plays an effective role in the healing of both remnants and transplanted lesions.^46^ Therefore, ARH is an alternative treatment for patients of SCC or AC/ASC CC. By contrast, after >1–2 cycles (generally 21 days per cycle) of NACT, disease progression can occur if patients are not sensitive to NACT. Moreover, NACT can lead to severe side effects of chemotherapy, with resulting delays and missed opportunities for optimal treatment, ultimately resulting in poor prognosis or even death.^47^ This may explain the worse outcomes we identified for NACT. Similarly, the low‐dose chemotherapy drugs used in R‐CT therapy may not be sufficient to reduce distant metastasis in AC/ASC CC, leading to poor oncological outcomes.^28^ Moreover, R‐CT can produce toxic side effects,^48^, ^49^, ^50^ having a negative impact on patients’ physical status. Considering that physical status positively correlates with treatment outcomes in patients with recurrent and metastatic disease and the patients with AC/ASC CC are prone to tumor recurrence and metastasis,^51^ R‐CT is likely to be less effective for the treatment of AC/ASC CC.

Previous studies predominantly used the FIGO 2009 tumor staging classification, included cases with positive and negative LN, and did not exclude patients treated laparoscopically. The main surgical procedures for CC are laparotomy, minimally invasive surgery, and robotic‐assisted surgery, yet there is still debate as to which approach is more advantageous.^52^, ^53^ In 2018, the report of the Laparoscopic Approaches to Cervical Cancer Treatment (LACC), a phase III randomized trial with a high level of evidence, showed that laparoscopic treatment of patients with CC had a worse prognosis for their tumors compared to open surgery.^54^ Accordingly, we limited our study sample to cases treated with laparotomy for CC with LNM, which may explain differences with findings in previous studies. Furthermore, because AC and ASC have similar clinical features and survival prognosis,^21^ we classified AC and ASC into the same group for analysis.

Our study has the advantage of including a high volume of cases using the 2018 FIGO classification to restrict the sample to stage IIIC CC. Additionally, oncological outcomes were compared for three initial treatments (R‐CT, ARH and NACT) based on the SCC and AC/ASC histological types. The constraints of our study also demand recognition. First, this study was retrospective and some heterogeneity of clinical data is inevitable, lack of information on specific regimens, doses, and duration of RT, chemotherapy, postoperative adjuvant therapy, and neoadjuvant therapy. Second, the lack of information on risk factors such as LVSI and parametrial involvement in the R‐CT group, and the fact that there were only 70 patients with AC/ASC in this group, may have affected the reliability of the results. Third, stratification was based on histologic type to exclude other influencing factors, such as lymph node status. Fourth, this study did not address aspects such as complications, toxic side effects, and the long‐term outcomes of the three treatment modalities.

In summary, initial treatment does influence the oncological prognosis of patients with stage IIIC CC, with added effects of the histological types of CC. ARH has shown efficacy in curing stage IIIC cervical SCC and AC/ASC and is an alternative treatment; however, caution needs to be exercised in the use of NACT, whereas R‐CT has no advantage in the treatment of AC/ASC.

## AUTHOR CONTRIBUTIONS

**Yanna Ye:** Conceptualization (lead); formal analysis (equal); methodology (lead); project administration (lead); writing – original draft (lead); writing – review and editing (lead). **Guochao zhang:** Conceptualization (equal); data curation (equal); investigation (equal); methodology (equal); project administration (equal); writing – original draft (equal); writing – review and editing (equal). **Zhiqiang Li:** Conceptualization (equal); data curation (equal); investigation (equal); methodology (equal); project administration (equal); writing – original draft (equal); writing – review and editing (equal). **Biliang Chen:** Conceptualization (equal); formal analysis (equal); investigation (equal); methodology (equal); writing – original draft (equal); writing – review and editing (equal). **Hongwei Zhao:** Formal analysis (equal); investigation (equal); writing – original draft (equal); writing – review and editing (equal). **Ying Yang:** Resources (equal); writing – original draft (equal). **Li Wang:** Resources (equal); writing – original draft (equal). **Jilong Yao:** Formal analysis (equal); methodology (equal); resources (equal); validation (equal). **Xiaolin Chen:** Data curation (equal); formal analysis (equal); investigation (equal); methodology (equal); validation (equal). **Yahong Huang:** Formal analysis (equal); investigation (equal); methodology (equal); validation (equal). **Jinghe Lang:** Conceptualization (equal). **Ping Liu:** Conceptualization (lead); supervision (lead). **Chunlin Chen:** Conceptualization (equal); supervision (equal).

## FUNDING INFORMATION

This study was funded by the National Science and Technology Support Program of China (Grant No. 2014BAI05B03), National Natural Science Fund of Guangdong (Grant No. 2015A030311024), Dongguan Sci‐tech Commissioner Program (Grant No. 20221800500562), and Science and Technology Plan of Guangzhou (Grant No. 158100075). [Correction added on October 18, 2023 after first online publication. The grant/award number for the Dongguan Sci‐tech Commissioner Program has been corrected in this version.]

## CONFLICT OF INTEREST STATEMENT

All the authors have completed the ICMJE uniform disclosure form. The authors declare no conflicts of interest. The authors are accountable for all aspects of this work and ensure that questions related to the accuracy or integrity of any part of the work are appropriately investigated and resolved.

## ETHICS STATEMENT

This study was conducted in accordance with the ethical principles of the Declaration of Helsinki 1964. Approval of the research protocol by an Institutional Reviewer Board: This retrospective study was approved by the Ethics Committee of the Nanfang Hospital of Southern Medical University (approval number NFEC‐2017‐135). Informed Consent: The IRB deemed that written informed consent was not necessary owing to the retrospective nature of the research and concealment of patient information. Registry and the Registration No. of the Study/Trial: Clinical trial number CHiCTR1800017778; International Clinical Trials Registry Platform Search Port, https://trialsearch.who.int/Trial2.aspx?TrialID=ChiCTR1800017778, registered at 14/08/2018. Animal Studies: N/A.

## Supporting information


Tables S1–S4.

## Data Availability

Data supporting the findings of this study are available upon request from the corresponding authors. The data are not publicly available because of privacy and ethical restrictions. These data are also related to unpublished studies. However, these data involve privacy issues for some patients and are not publicly shared.
