# Supplementary material for: Initial treatment for FIGO 2018 stage IIIC cervical cancer based on histological type: A 14‐year multicenter study
Source: Cancer Med. 2023 Sep 28;12(19):19617–32. doi: 10.1002/cam4.6586 (PMC10587947; doi:10.1002/cam4.6586)
Supplement: Supplementary file 1 — Tables S1–S4. [file CAM4-12-19617-s001.docx]

**Table S1 Clinicopathological characteristics of patients with SCC and AC/ASC**

| **Variables** | **Before PSM** | | | **After PSM** | | |
| --- | --- | --- | --- | --- | --- | --- |
|  | **SCC group**  (3761) % | **AC/ASC group**  (325) % | P | **SCC group**  (2197) % | **AC/ASC group**  (321) % | P |
| **Age (years)** | 51.07±10.516 | 49.51±10.575 | 0.010 | 49.92±10.051 | 49.55±10.216 | 0.534 |
| **Initial treatment modality** |  |  | ＜0.001 |  |  | 0.230 |
| R-CT | 1843(49.0%) | 70(21.5%) |  | 1188(54.1%) | 171(53.3%) |  |
| ARH | 1355(36.0%) | 174(53.6%) |  | 545(24.8%) | 70(21.8%) |  |
| NACT | 563(15.0%) | 81(24.9%) |  | 464(21.1%) | 80(24.9%) |  |

*FIGO: International Federation of Gynecology and Obstetrics; CC: cervical cancer; SCC: squamous cell carcinoma; AC: adenocarcinoma; ASC: adenosquamous cell carcinoma;* *R-CT: radical chemoradiotherapy; ARH: abdominal radical hysterectomy; NACT: neoadjuvant chemotherapy and radical surgery.*

**Table S2 Clinicopathological characteristics of patients with SCC and AC/ASC in the R-CT group**

| **Variables** | **SCC group**  **(n=1843)** | **AC/ASC group**  **(n=70)** | **P** |
| --- | --- | --- | --- |
| **Age (years)** | 54.53±10.791 | 55.83±11.036 | 0.324 |

*SCC: squamous cell carcinoma; AC: adenocarcinoma; ASC: adenosquamous cell carcinoma;* *R-CT: radical chemoradiotherapy*

**Table S3 Clinicopathological characteristics of patients with SCC and AC/ASC in the ARH group**

| **Variables** | **Before PSM** | | | **After PSM** | | |
| --- | --- | --- | --- | --- | --- | --- |
|  | **SCC group**  (n=1355) | **AC/ASC group**  (n=174) | P | **SCC group**  (n=344) | **AC/ASC group**  (n=173) | P |
| **Age (years)** | 48.24±9.A | 48.02±9.854 | 0.773 | 47.82±9.267 | 47.97±9.863 | 0.867 |
| **Hysterectomy type** |  |  | 0.240 |  |  | 0.859 |
| Type QM-B | 964 (71.1%) | 113 (64.9%) |  | 229 (66.6%) | 113 (65.3%) |  |
| Type QM-C1 | 6 (0.4%) | 1 (0.6%) |  | 114 (33.1%) | 59 (34.1%) |  |
| Type QM-C2 | 385 (28.4%) | 60 (34.5%) |  | 1 (0.3%) | 1 (0.6%) |  |
| **Tumor diameter（cm）** |  |  | 0.748 |  |  | 0.974 |
| ≤4 | 914 (67.5%) | 113 (64.9%) |  | 228 (66.3%) | 113 (65.3%) |  |
| ＞4 | 397 (29.3%) | 54 (31.0%) |  | 102 (29.7%) | 53 (30.6%) |  |
| Unknown | 44 (3.2%) | 7 (4.0%) |  | 14 (4.1%) | 7 (4.0%) |  |
| **LVSI** |  |  | <0.001 |  |  | 0.914 |
| negative | 731 (53.9%) | 130 (74.7%) |  | 255 (74.1%) | 129 (74.6%) |  |
| positive | 624 (46.1%) | 44 (25.3%) |  | 89 (25.9%) | 44 (254%) |  |
| **Cervical stromal invasion** |  |  | 0.680 |  |  | 0.853 |
| ≤1/2 | 184 (13.6%) | 24 (13.8%) |  | 47 (13.7%) | 24 (13.9%) |  |
| ＞1/2 | 1126 (83.1%) | 142 (81.6%) |  | 277 (80.5%) | 141 (81.5%) |  |
| Unknown | 45 (3.3%) | 8 (4.6%) |  | 20 (5.8%) | 8 (4.6%) |  |
| **Parametrial involvement** |  |  | 0.551 |  |  | 0.502 |
| Negative | 1284 (94.8%) | 163 (93.7%) |  | 327 (95.1%) | 162 (93.6%) |  |
| Positive | 71 (5.2%) | 11 (6.3%) |  | 17 (4.9%) | 11 (6.4%) |  |
| **Vaginal margin** |  |  | 0.949 |  |  | 0.586 |
| Negative | 1307 (96.5%) | 168 (96.6%) |  | 335 (97.4%) | 167 (96.5%) |  |
| Positive | 48 (3.5%) | 6 (3.4%) |  | 9 (2.6%) | 6 (3.5%) |  |
| **Para-aortic lymph node** |  |  | 0.217 |  |  | 0.974 |
| Negative | 183 (3.5%） | 27 (15.5%) |  | 52 (15.1%) | 27 (15.6%) |  |
| Positive | 29 (2.1%) | 7 (4.0%) |  | 11 (3.2%) | 6 (3.5%) |  |
| Unknown | 1143 (84.4%) | 140 (80.5%) |  | 281 (81.7%) | 140 (80.9%) |  |

*SCC: squamous cell carcinoma; AC: adenocarcinoma; ASC: adenosquamous cell carcinoma;* *ARH: abdominal radical hysterectomy; LVSI: lymphovascular space invasion.*

**Table S4 Clinicopathological characteristics of patients with SCC and AC/ASC in the NACT group**

| **Variables** | **Before PSM** | | | **After PSM** | | | |
| --- | --- | --- | --- | --- | --- | --- | --- |
|  | **SCC group**  (n=563) | **AC/ASC group**  (n=81) | P | **SCC group**  (n=153) | **AC/ASC group**  (n=77) | | P |
| **Age (years)** | 48.24±9.357 | 48.02±9.854 | 0.773 | 46.35±8.398 | 47.56±8.819 | 0.313 | |
| **Hysterectomy type** |  |  | 0.087 |  |  | 0.500 | |
| Type QM-B | 377 (67.0%) | 64 (79.0%) |  | 113 (73.9%) | 60 (77.9%) |  | |
| Type QM-C1 | 184 (32.7%) | 17 (21.0%) |  | - | - |  | |
| Type QM-C2 | 2 (0.4%) | 0 (0.0%) |  | 40 (26.1%) | 17 (22.1%) |  | |
| **Tumor diameter (cm)** |  |  | 0.695 |  |  | 0.938 | |
| ≤4 | 182 (32.3%) | 30 (37.0%) |  | 55 (35.9%) | 27 (35.1%) |  | |
| ＞4 | 326 (57.9%) | 44 (54.3%) |  | 88 (57.5%) | 44 (57.1%) |  | |
| Unknown | 55 (9.8%) | 7 (8.6%) |  | 10 (6.5%) | 6 (7.8%) |  | |
| **LVSI** |  |  | 0.739 |  |  | 0.977 | |
| Negative | 400 (71.0%) | 59 (72.8%) |  | 111 (72.5%) | 56 (72.7%) |  | |
| Positive | 163 (29.0%) | 22 (27.2%) |  | 42 (27.5%) | 21 (27.3%) |  | |
| **Cervical stromal invasion** |  |  | 0.275 |  |  | 0.542 | |
| ≤1/2 | 102 (18.1%) | 9 (11.1%) |  | 24 (15.7%) | 8 (10.4%) |  | |
| ＞1/2 | 430 (76.4%) | 68 (84.0%) |  | 124 (81.0%) | 66 (85.7%) |  | |
| Unknown | 31 (5.5%) | 4 (4.9%) |  | 5 (3.3%) | 3 (3.9%) |  | |
| **Parametrial involvement** |  |  | 0.025 |  |  | 0.437 | |
| Negative | 526 (93.4%) | 70 (86.4%) |  | 140 (91.5%) | 68 (88.3%) |  | |
| Positive | 37 (6.6%) | 11 (13.6%) |  | 13 (8.5%) | 7 (11.7%) |  | |
| **Vaginal margin** |  |  | 0.115 |  |  | 0.806 | |
| Negative | 547 (97.2%) | 76 9 (3.8%) |  | 148 (96.7%) | 74 (96.1%) |  | |
| Positive | 16 (2.8%) | 5 (6.2%) |  | 5 (3.3%) | 3 (3.9%) |  | |
| **Para-aortic lymph node** |  |  | 0.240 |  |  | ＜0.001 | |
| Negative | 79 (14.0%) | 13 (16.0%) |  | 5 (3.3%) | 57 (74.0%) |  | |
| Positive | 16 (2.8%) | 5 (6.2%) |  | 0 (0%) | 20 (26%) |  | |
| Unknown | 468 (83.1%) | 63 (77.8%) |  | 148 (96.7%) | 0 (0%) |  | |

*SCC: squamous cell carcinoma; AC: adenocarcinoma; ASC: adenosquamous cell carcinoma;*  *NACT: neoadjuvant chemotherapy and radical surgery; LVSI: lymphovascular space invasion.*
